# Emergency Laparotomy Follow-Up Study (ELFUS): prospective feasibility investigation into postoperative complications and quality of life using patient-reported outcome measures up to a year after emergency laparotomy

**DOI:** 10.1186/s13741-021-00193-5

**Published:** 2021-07-26

**Authors:** D. I. Saunders, R. C. F. Sinclair, B. Griffiths, E. Pugh, D. Harji, B. Salas, H. Reed, C. Scott

**Affiliations:** 1 Department of Anaesthesia, Royal Victoria Infirmary, Newcastle upon Tyne, NHS FT, NE1 4LP UK; 2 Department of Colorectal Surgery, Royal Victoria Infirmary, Newcastle upon Tyne, NHS FT, NE1 4LP UK; 3 Newcastle University, Newcastle upon Tyne, NE2 4HH UK; 4 Research Nurse, Department of Research and Development, Royal Victoria Infirmary, Newcastle upon Tyne, NHS FT, NE1 4LP UK

**Keywords:** Quality of life; Emergency laparotomy, Complications, Patient-reported outcome measures (PROMS)

## Abstract

**Background:**

Emergency laparotomy carries a significant risk profile around the time of surgery. This research aimed to establish the feasibility of recruitment to a study using validated scoring tools to assess complications after surgery; and patient-reported outcome measures (PROMs) to assess quality of life and quality of recovery up to a year following emergency laparotomy (EL).

**Methods:**

We used our local National Emergency Laparotomy Audit (NELA) register to identify potential participants at a single NHS centre in England. Complications were assessed at 5, 10 and 30 days after EL. Patient-reported outcome measures were collected at 1, 3, 6 and 12 months after surgery using EQ5D and WHODAS 2.0 questionnaires.

**Results:**

Seventy of 129 consecutive patients (54%) agreed to take part in the study. Post-operative morbidity survey data was recorded from 63 and 37 patients at postoperative day 5 and day 10. Accordion Complication Severity Grading data was obtained from 70 patients. Patient-reported outcome measures were obtained from patients at baseline and 1, 3, 6 and 12 months after surgery from 70, 59, 51, 48, to 42 patients (100%, 87%, 77%, 75% and 69% of survivors), respectively.

**Conclusions:**

This study affirms the feasibility of collecting PROMs and morbidity data successfully at various time points following emergency laparotomy, and is the first longitudinal study to describe quality of life up to a year after surgery. This finding is important in the design of a larger observational study into quality of life and recovery after EL.

**Supplementary Information:**

The online version contains supplementary material available at 10.1186/s13741-021-00193-5.

## Introduction

Major non-elective abdominal surgery (“emergency laparotomy, EL”) leads to a long hospital admission and carries a significant risk of mortality at 30 and 90 days after surgery (9.6% and 12.9% respectively) (National Emergency Laparotomy Audit 2019). Patients are frequently elderly (45% age 70 or above), have co-morbidity (over half are graded ASA3 or above), and present in an acutely unwell condition (over half are deemed to have a predicted risk of mortality of over 5%) (National Emergency Laparotomy Audit 2019). The development of a mandatory data registry, the National Emergency Laparotomy Audit (NELA), has enabled systematic recording of process and outcome measures for patients undergoing EL in England and Wales since 2013. The most recent annual report from NELA analysed 24000 patients from 179 hospitals who underwent emergency surgery in 2018 (National Emergency Laparotomy Audit 2019). Despite significant improvements since the first UK report into patient outcomes (Saunders et al. 2012), mortality remains high at 9.6%, with a prolonged median length of hospital stay of 16 days. NELA has a contractual obligation to limit the burden of data-entry for clinicians, and the outcomes dataset within the registry is restricted to short-term findings only. Despite the potential importance to patients and their families, detail about early post-operative morbidity, or the impact of EL on a patient’s longer-term functional ability and quality of life (QoL) is recorded in NELA at best in low fidelity, or not at all.

Patient-reported outcome data provides a more balanced and patient-centric perspective of treatment effects and their potential benefits beyond traditional clinical outcomes. Patient-reported outcome measures (PROMs) are routinely and successfully collected in the NHS in the elective setting in a number of conditions including hip and knee arthroplasty [NHS Digital Patient Reported Outcome Measures, 2020]. Collection of PROMs in the emergency setting is potentially challenging given the acute nature of disease presentation combined with deranged physiology and the time-sensitive need to deliver definitive treatment. Heterogeneity of disease presentation and duration, coupled with the immediacy of the clinical setting makes accurate capture of baseline QoL assessment difficult.

The current data about QoL following EL is limited to a few studies, with little robust data available. Stevens et al. described variable PROMs reporting in clinical trials conducted in the emergency setting, with the use of multiple outcome measures, limited baseline data and a lack of a priori hypotheses (Stevens et al. 2016). Blazeby et al. studied the feasibility of collecting PROMs in the emergency setting, collecting data on all patients presenting with emergency pathology irrespective of treatment modality (Mason et al. 2015). This group reported good baseline compliance; but high rates of attrition led to limited reporting of longitudinal data (Mason et al. 2015). Kwong et al. investigated the feasibility of collecting cross-sectional retrospective generic and disease specific QoL data in patients who had been registered in NELA (Kwong et al. 2018). They recruited 268 patients from 13 hospitals and made contact with 255 survivors 3 months after surgery. The overall response rate was 74.1%, adding to the emerging evidence base about the feasibility of this approach in the emergency setting (Kwong et al. 2018). The same group also studied their ability to collect retrospective baseline PROMs in medical emergencies (acute myocardial infarction) (Kwong and Black 2018). Ninety percent of those invited to participate agreed to take part, although variation in approaching eligible patients in different hospitals led to recruitment bias. Given the high response rate demonstrated by this group, this may be considered a potentially suitable option in EL patients.

There is a complex interplay between quality of life (QoL) and post-operative clinical outcomes in surgery. Post-operative complications are associated with prolonged recovery, increased length of stay and have an adverse impact on all aspects of QoL (Khuri et al. 2005). Despite its importance to patients and their families, post-operative morbidity is poorly reported in the emergency setting, with the focus being on post-operative mortality. We believe it is important to document post-operative morbidity in patients undergoing EL and to investigate the relationship between morbidity and subsequent quality of life. The aims of the Emergency Laparotomy Follow-Up Study (ELFUS) were to assess the feasibility of collecting accurate, longitudinal QoL data and morbidity in the setting of EL during the first year of recovery prior to considering a definitive, larger-scale future study.

## Methods

The study protocol was approved by the Newcastle and North Tyneside Regional Ethics Committee (16/NE/0334) and sponsored by Newcastle upon Tyne Hospitals NHS Foundation Trust. ELFUS was designed as a prospective feasibility observational study of in-hospital postoperative complications and post-discharge patient reported outcome measures following emergency laparotomy. Adult patients were recruited during a 7-month period at the Royal Victoria Infirmary (RVI), Newcastle, and followed up for a year after surgery. Eligibility criteria were that the patient had undergone surgery that met inclusion criteria for NELA and that patient data had been entered into our local NELA registry (NELA inclusion criteria, 2013).

Patients or their relatives/advocates were approached during the first 4 days after EL in either critical care or the general surgery postoperative ward. Written informed consent was taken from each participant, or alternatively from their proxy in the situation where sedation and ventilation prevented capacity to give consent. Participant consent was gained retrospectively for these individuals once capacity was regained.

The World Health Organisation Disability Schedule 2.0 (WHODAS 2.0) [World Health Organisation, 2018] and the EuroQol Five Dimensions (EQ5D) [Euroqual Research Foundation, 2019] questionnaires were used to assess QoL. Study participants in hospital at the relevant timepoints were offered paper copies of the questionnaires, given whatever time they required, and asked to circle their most appropriate answers using a pen. For those who required assistance, and for those who had left hospital, a member of the research team read out the questions either directly at the bedside, or over the telephone, and recorded answers on paper on behalf of the participant. Baseline data were collected at day 5, and longitudinal data were collected at 1, 3, 6 and 12 months post-operatively. The Post-Operative Morbidity Survey (POMS) (Grocott et al. 2007) and the Accordion Severity Grading Classification System (Strasberg et al. 2009) were used to assess post-operative morbidity. POMS assessments were undertaken at postoperative day 5 and day 10; and the Accordion Severity Grading was assessed at 30 days after surgery. A schedule of assessments of complications and QoL at various timepoints is shown in Table 1. See Additional files 1 and 2 for detail of complications and QoL assessment tools.

**Table 1:** Schedule of patient assessments

Timepoint (post-laparotomy)	Action/assessments
Within 4 days of EL (day 0-4)	Identification of potential study participants Distribution of participant information sheet Informed consent taken
Day 5	Baseline assessment of QoL^a^: WHODAS^a^ and EQ5D^a^ POMS^a^
Day 10	POMS for those still in-patient
Day 30	QoL assessment with WHODAS and EQ5D Accordion Severity Grading Assessment
3 months	QoL assessment with WHODAS and EQ5D
6 months	QoL assessment with WHODAS and EQ5D
12 months	QoL assessment with WHODAS and EQ5D

^a^*QoL* means quality of life, *WHODAS* means World Health Organisation disability assessment score 2.0, *EQ5D* means 5-domain Euroqual quality of life assessment, *POMS* means Post-Operative Morbidity Survey

### Primary endpoint

Our primary endpoint was feasibility of PROMs and post-operative complication data collection following EL. This was assessed using data compliance, calculated as the proportion of completed questionnaires received at each time point.

### Statistics and analytical methods

The target sample size of 70 patients was determined to allow precise estimation of parameters of interest. Previously, sample sizes of 24–50 have been recommended for use in clinical feasibility studies (Sim and Lewis 2012). The Shapiro-Wilk test was used to confirm non-parametric distribution of patient characteristics. Continuous non-parametric variables were compared using Mann-Whitley *U* tests. Categorical data were analysed using Fisher’s exact test. Minitab 18 software was used for statistical testing.

## Results

### Patient recruitment

**Fig. 1:**
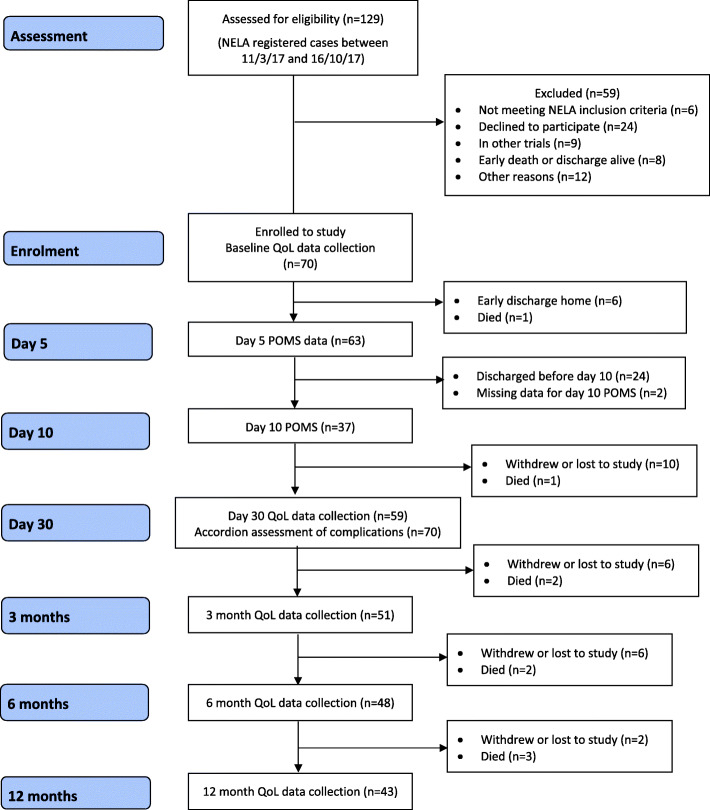
Participant flow diagram

Of 129 patients screened and assessed for eligibility, 70 patients agreed to participate (recruitment rate of 54.4%). Twenty-four patients (18.5%) declined to take part, and 35 were considered ineligible. Reasons for this included not fulfilling NELA inclusion criteria (6 patients); participation in other trials (9 patients); and early discharge from hospital or early death after surgery (8 patients) (Fig. 1). Baseline patient and clinical characteristics are shown in Table 2. Study participants had lower predicted median P-POSSUM and NELA mortality risks compared to those who were not recruited (4.6% vs 8.8% and 3.3% vs 4.1% respectively).

**Table 2:** NELA registrant and study participant characteristics. Values are number (proportion) or median (IQR [range])

	All registrants in NELA during study period (***n*** = 129)	Study participants (***n*** = 70)	Excluded from study (***n*** = 59)	***p*** value
Age	66 (50.5–76.5 [21.0–89])	65.5 (50–76 [21–89])	66 (56–77 [22–88])	0.574
P-Possum mortality risk pre-op (%)	5.7 (2.6–20.1 [0.7–98.5])	4.6 (1.8–15.6 [0.7–83.9])	8.8 (4.1–31.8 [0.8–98.5])	0.006
P-Possum morbidity risk pre-op (%)	69.0 (47.5–90.6 [17.5–100])	62.1 (39.2–86.0 [17.5–99.4])	83.5 (59.1–94.5 [19.9–100])	0.004
NELA mortality risk pre-op (%)	3.7 (1.2–11.6 [0.1–88.9])	3.3 (0.9–10.0 [0.1–61.3])	4.1 (1.6–18.9 [0.3–88.9])	0.094
ASA physical status	3 (2–3 [1–5])	3 (2–3 [1–5])	3 (2–3 [1–5])	0.086
Female patient	55 (42.6)	33 (47.1)	22 (37.3)	0.287
Critical care stay (days)	2 (2–4 [0–52])	2 (2–4 [0–15])	2 (2–5 [2–52])	0.383
Return to theatre	10 (7.8)	6 (8.6)	4 (6.8)	0.754
Postop LOS hospital (days)^a^	9.8 (5.5–19.4 [0–60])	9.3 (6.0–23.7 [0–60])	11.3 (5.4–17.9 [0–60])	0.919
Observed In-hospital mortality	13 (10.1)	4 (5.7)	9 (15.3)	0.085

^a^ Length of postoperative stay assessment capped at 60 days

### Data compliance PROMs and post-operative morbidity

Data compliance with WHODAS 2.0 and EQ-5D questionnaires at baseline and at follow-up timepoints is shown in Table 3. Baseline data collected on day 5 post-operatively had a compliance rate of 100% for PROMS and post-operative morbidity. Data compliance at 12 months for both WHODAS 2.0 and EQ-5D were 69%. Data compliance for the assessment of post-operative morbidity using the POMS and Accordion Severity Classification were at least 95%.

**Table 3:** Number of respondents to PROMs questionnaires at different time points

	Baseline/enrolment	1 month	3 months	6 months	12 months
Possible respondents (survivors)	70	68	66	64	61
EQ5D completed questionnaires	70	59 (87%)	51 (77%)	48 (75%)	42 (69%)
WHODAS completed questionnaires	70	59 (87%)	51 (77%)	48 (75%)	42 (69%)
WHODAS score median (IQR) [range]	3 (1–14) [0–36]	14 (8–23) [0–38]	6 (1–18) [0–35]	4.5 (0–12.5) [0–33]	2 (0–19) [0–38]
Number (proportion) with WHODAS score of zero	14 (20.0%)	4 (6.8%)	9 (17.6%)	14 (29.2%)	13 (31.7%)

Proportion are numbers of respondents completing questionnaires fully compared to number of possible respondents (survivors)

### WHODAS 2.0

WHODAS disability scores were calculated for 70 patients at baseline and 42 patients at 12 months. Fourteen (20.0%) patients were found to have no disability at baseline. At 12-month follow-up, 13 (31.7%) patients were found to have no disability (Table 3). The observed median WHODAS scores across all candidate timepoints were between 3 and 14. Please see Additional file 3 for detail about respondents to various WHODAS domains at candidate time points.

### EQ5D

EQ5D scores were calculated for 70 patients at baseline and 42 patients at 12 months (Table 3). Across all domains, EQ5D identified health problems at baseline, with observed rates of 58.6% in the pain domain, 47.1% in the anxiety domain and 41.4% in the ability to perform usual activities domain. The EQ-5D was able to identify health problems at all candidate timepoints with response rates amongst survivors of 87%, 77%, 75% and 70% at 1, 3, 6 and 12 months post-operatively. Please see Additional file 4 for detail about study participants who responded to EQ5D questionnaire domains at candidate time points. Additional file 5 shows the proportion of respondents reporting EQ5D levels 1–5 at candidate time points; and Additional file 6 shows the proportion of respondents with problems or no problems at candidate time points.

### Postoperative complications data

**Fig. 2:**
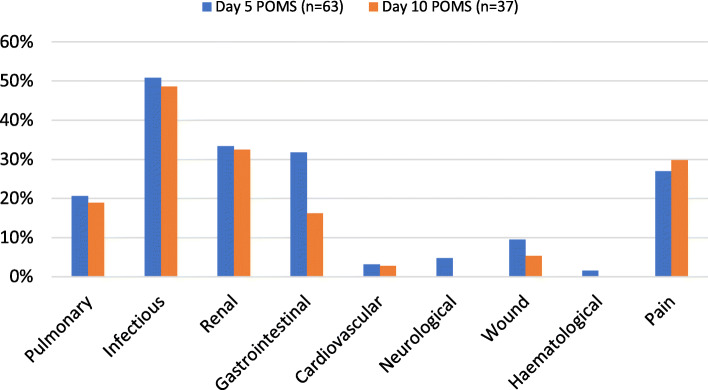
Distribution of POMS positive domains for patients remaining inpatient at day 5 and 10 after EL

Six patients were discharged from hospital within 5 days, and one patient died. POMS scores were collected for all 63 remaining in-patients (90% of study participants) at post-operative day 5. Thirty-nine patients remained in hospital at day 10, and POMS scores were collected for 37 (52.8% of study participants). A POMS score of 0 was observed for 15/63 (23.8%) patients on day 5, and 14/37 (37.8%) on day 10. A breakdown of the types of complications observed is outlined in Fig. 2.

Accordion Severity Grading of complications were collected for all 70 patients who participated in the study. No complications were observed in 39 (55.7%) patients. Complications of grade 3 severity and above were observed in 19 (27.1%) patients.

## Discussion

The ELFUS study demonstrates that it is feasible to collect valid and accurate QoL and complication data in patients who have undergone emergency laparotomy.

We were able to recruit 54% of patients undergoing EL at our institution, and accurately collect baseline and longitudinal 12-month QoL data, with response rates of 100% and 70% respectively. Alongside this, we were able to collect accurate and validated 30-day morbidity data in all patients. An important finding in our study is around recruitment bias, with the study cohort expressing less physiological derangement and sickness severity compared to those not recruited, as demonstrated by lower predicted morbidity and mortality risk estimates at the time of surgery.

Previous reports of collecting longitudinal QoL data in the emergency setting have demonstrated high attrition rates at 12 months. Kwong et al. were able to demonstrate response rates of 70% at 3 months post-operatively, but they did not extend data collection beyond this timepoint (Kwong et al. 2018). Our study reports similar early response rates but demonstrates a consistent response rate of 70% at 12 months. Pre-operative baseline data needs to be reflective of the health status of patients prior to emergency laparotomy. Collecting this data prior to surgery can be difficult due to the time-sensitive need to deliver definitive clinical care. Consequently, we collected our baseline data at 5 days post-operatively. Using this approach, we were able to recruit 54% of our emergency laparotomy population and collect 100 percent of baseline QoL data. PROMs questionnaires such as WHODAS 2.0 and EQ5D capture QoL over a period of 30 days, so the collection of baseline data at day 5 post-operatively should adequately capture pre-operative QoL and overall health status, prior to assessing the impact of emergency laparotomy.

Morbidity is considered to be an important outcome measure in emergency laparotomy with the widely used Portsmouth Physiological and Operative Severity Score for the enumeration of Mortality and Morbidity (P-POSSUM) scoring system providing predicted post-operative morbidity scores for patients as a part of the pre-operative risk stratification process (Prytherch et al. 1998). Despite its emphasis, morbidity is poorly reported as a robust outcome measure in emergency laparotomy, with a varying documented incidence of 33–71% in the literature (Tengberg et al. 2017; Tolstrup et al. 2017). This may be due to variable outcome reporting, a lack of appropriate definitions and the failure to use available standardised scoring systems. We employed the POMS scoring system to facilitate data collection and to appropriately categorise complications; and the Accordion Severity Scoring System to grade complications. Employing two scoring systems enabled us to categorise complications in 100% of inpatients on day 5 and 94.9% of inpatients on day 10 (90% and 52.8% of study participants respectively), whilst providing Accordion Severity data in all patients recruited into our study. A modified version of POMS scoring system has been previously used in the emergency setting to assess outcomes over multiple timepoints within a 30-day timeframe. Howes et al. identified that POMS-defined morbidity was highest on day 3 and reduced at each successive time-point (Howes et al. 2015). This reflects our own experience and suggests early collection of inpatient morbidity is key to good outcome reporting of post-operative morbidity. The use of the POMS system in the emergency setting is simple and effective; and should be employed in the early post-operative period. Ensuring consistency in the use of these scoring systems is in line with recent consensus recommendations to include outcome measures so that pooling and meta-analysis of results is facilitated (Myles et al. 2018, Shulman et al. 2015). A larger study using the measures should permit direct comparison of outcomes for EL patients with other patient groups, as well as provide robust information about quality of recovery for EL patients.

Outcome assessment in the emergency setting has begun to look beyond 30- and 90-day mortality to include broader patient-related factors including pre-operative frailty. The Emergency Laparotomy and Frailty (ELF) group demonstrated that the presence of pre-operative frailty was associated with a greater risk of post-operative mortality and morbidity (Parmar et al. 2019). Routine collection of frailty scores have been introduced into the current NELA dataset (National Emergency Laparotomy Audit 2019). Outcome reporting in the emergency setting should be expanded further to include QoL, with a particular emphasis on physical impact, functional impairment and the presence or lack of disability. QoL is an important outcome measure in its own right, but also of importance is a need to measure freedom from impairment or disability, especially as the very point of surgery is to relieve suffering and to cure a state of disease (Shulman et al. 2015). Disability-free survival is an attractive outcome measure for studies of the impact of surgery, as it reflects the primary goal for most patients and has been recommended as a meaningful endpoint in studies of surgical patients (Chalmers et al. 2001). This outcome measure could be used to help develop and refine rehabilitation and recovery programmes in the future, as well as informing pre-operative discussions around the time of emergency laparotomy (Glance et al. 2014). WHODAS 2.0 is measure of physical function and disability and has been used to define disability-free survival after surgery (Shulman et al. 2015). Using WHODAS 2.0, we were able to identify functional impairment in 56 (80.0%) patients at baseline and 30 (69.7%) patients at 12 months. The measure was able to identify patients with a range of scores, which reflects differing levels of disability. The wider utility of this measure in the emergency surgery setting needs further investigation, to assess the definitive impact of emergency laparotomy on functional and physical outcomes amongst patient groups of varying age, co-morbidity, pathology and sickness severity at the time of surgery.

The recruitment bias in our study is an important consideration in future work. Recruiting a truly representative cohort of emergency surgery patients is difficult given overriding clinical priorities in the acute and initial phases of their clinical course. Further work must be undertaken to identify how this critically unwell population cohort can be included into emergency surgery research frameworks to ensure wider generalisability. At the inception of NELA, exemption for patient consent was granted under section 251 of the 2006 NHS act, acknowledging that recruiting high risk patients into the registry at a time of acute illness would be problematic were consent to be required. One possible solution to the recruitment bias we experienced in our study would be to add additional patient-centric outcome measures to the dataset of NELA, perhaps in a representative subset of participating hospitals. Collection of baseline QoL data is an important methodological consideration in emergency surgery research: employing a baseline timepoint following the immediate acute event may potentially enable collection of QoL in a greater proportion of patients. Timing of ‘baseline’ data collection in the emergency setting needs further research.

## Conclusions

The ELFUS study affirms the feasibility of collecting PROMs and morbidity data successfully at a number of time points following surgery. This is the first longitudinal study to describe PROMs and QoL up to a year following emergency general abdominal surgery. The ELFUS study demonstrates that it is possible to capture baseline PROMS data in the emergency setting by changing the timing of the initial assessment, whilst demonstrating low rates of attrition for longitudinal assessments. Post-operative morbidity data can be defined and graded appropriately and collected in the emergency setting when standardised scoring systems are employed. These methodological considerations can be employed in a larger study to collect PROMs and morbidity data and to assess the impact of emergency laparotomy on these important outcomes. Further work is needed to assess the optimum timing of baseline assessments of QoL. Any future research should consider how to reduce recruitment bias, ensuring all emergency laparotomy patients are included in assessments of the impact of surgery on their recovery and subsequent quality of life.

## Supplementary Information

**Additional file 1:.** Complication scoring and assessment tools.

**Additional file 2:.** Quality of life assessment tools.

**Additional file 3:.** Number of respondents to WHODAS domains at candidate time points (landscape table only).

**Additional file 4:.** Number of respondents to EQ5D domains at candidate follow up timepoints (portrait table only).

**Additional file 5:.** Additional file 5: Proportion of respondents reporting levels 1-5 in EQ5D at candidate follow up points (portrait table only).

**Additional file 6:.** Additional file 6: Respondents reporting EQ5D problems or no problems at candidate follow up points (portrait table only).

## Data Availability

The datasets used and analysed during this study are stored securely in our institution and available from the corresponding author on reasonable request.
