# Supplementary material for: Emergency Laparotomy Follow-Up Study (ELFUS): prospective feasibility investigation into postoperative complications and quality of life using patient-reported outcome measures up to a year after emergency laparotomy
Source: Perioper Med (Lond). 2021 Jul 26;10:22. doi: 10.1186/s13741-021-00193-5 (PMC8311937; doi:10.1186/s13741-021-00193-5)
Supplement: Supplementary file 1 — Additional file 1:. Complication scoring and assessment tools. [file 13741_2021_193_MOESM1_ESM.docx]

## ELFUS additional material 1: complication scoring and assessment tools

## POMS

The post-operative morbidity survey (POMS) is a prospective method for describing short-term morbidity after major surgery (Grocott et al 2007). POMS was designed to be easy to perform and aims to identify only those morbidities that prevent discharge from hospital. A POMS score was calculated on day 5 and day 10 after EL using a combination of patient questioning, observation charts and case note review. POMS scoring does not take into account the *severity* of complications. Rather scores of 0 or 1 (absent or present) are assigned within each of the nine domains (pulmonary, infectious, renal, gastrointestinal, cardiovascular, neurological, haematological, wound and pain); giving an aggregate score out of nine.

## Accordion Classification of Complications

The Accordion Severity Grading System provides a meaningful approach to classifying complications after surgery according to the resources required in their management. Complications are graded from zero to 6 as described in the table below (Strasberg et al 2009). We assessed patients for the presence or absence of complications as per the Accordion Classification at 30 days after surgery.

***Accordion Classification of Post-operative Complications. Description of grades.***

| **Accordion Severity Grading of Complications** | **Description** |
| --- | --- |
| Zero | No complications |
| 1 (Mild) | Requires minor bedside management only eg insertion of cannula, or nasogastric tube, or drainage of superficial wound infection |
| 2 (Moderate) | Requires pharmacological treatment with drugs other than those allowed for minor complications such as antibiotics. Blood transfusion and/ or total parenteral nutrition are included |
| 3 (Severe: invasive procedure/ No GA) | Requires management by endoscopic therapy, an interventional procedure or re-operation without general anaesthesia |
| 4 (Severe: invasive procedure under GA, or single organ system failure) | Requires operation under general anaesthesia, or results in single organ dysfunction |
| 5 (Severe: multiple organ system dysfunction) | Such complications will usually require management in an intensive care unit. |
| 6 (Death) | Postoperative death |
