# Supplementary material for: Emergency Laparotomy Follow-Up Study (ELFUS): prospective feasibility investigation into postoperative complications and quality of life using patient-reported outcome measures up to a year after emergency laparotomy
Source: Perioper Med (Lond). 2021 Jul 26;10:22. doi: 10.1186/s13741-021-00193-5 (PMC8311937; doi:10.1186/s13741-021-00193-5)
Supplement: Supplementary file 2 — Additional file 2:. Quality of life assessment tools. [file 13741_2021_193_MOESM2_ESM.docx]

## ELFUS additional material 2: quality of life assessment tools

## EQ5D European Quality of Life Survey

EQ5D is a standardised measure of health status developed by the EuroQol group in order to provide a simple generic measure of health [Euroqual Research Foundation]. It assesses QoL in five domains (mobility, self-care, participation in usual activities, pain/ discomfort and anxiety/ depression). Each EQ5D domain is reported at five levels (1-5): level 1 indicates that there are no reported problems in this domain; whilst levels 2-5 show increasing severity of reported problems.

## WHO Disability Assessment Schedule 2.0 (WHODAS 2.0)

WHODAS 2.0 measures the limitations to activity and participation experienced by an individual as a result of a medical condition [World Health Organisation]. This tool includes six domains that allow assessment of understanding and communicating (cognition); getting around (mobility); self-care; getting along and interacting with other people; life activities such as domestic responsibilities and leisure; and participation in society.

We employed the WHODAS 2.0 twelve-point short-form assessment version of WHODAS 2.0. This assigns a score of zero to 4 (none, mild, moderate, severe, extreme) for each domain, and ultimately gives a simple sum score between 0 and 48, where 0 is no disability and 48 is full/severe disability. A WHODAS 2.0 score was calculated for each participant at each of the follow-up time points after discharge from hospital.
