# Supplementary material for: Emergency Laparotomy Follow-Up Study (ELFUS): prospective feasibility investigation into postoperative complications and quality of life using patient-reported outcome measures up to a year after emergency laparotomy
Source: Perioper Med (Lond). 2021 Jul 26;10:22. doi: 10.1186/s13741-021-00193-5 (PMC8311937; doi:10.1186/s13741-021-00193-5)
Supplement: Supplementary file 3 — Additional file 3:. Number of respondents to WHODAS domains at candidate time points (landscape table only). [file 13741_2021_193_MOESM3_ESM.docx]

***Additional file 3: Number of respondents (proportion) for WHODAS domains at candidate follow up timepoints***

| Time point | Number of possible respondents (survivors) | Respondents to WHODAS domains | | | | | | | | | | | |
| --- | --- | --- | --- | --- | --- | --- | --- | --- | --- | --- | --- | --- | --- |
|  |  | Standing | Household Responsibilities | New Task | Activities | Emotions | Concentrating | Walking | Washing | Dressing | New People | Friendships | Work |
| Baseline | 70 | 70 (100) | 70 (100) | 70 (100) | 70 (100) | 70 (100) | 70 (100) | 70 (100) | 70 (100) | 70 | 70 | 70 | 70 |
| 1 month | 68 | 59 (86.7) | 59 (86.7) | 59 (86.7) | 59 (86.7) | 58 (85.3) | 59 (86.7) | 59 (86.7) | 59 (86.7) | 59 | 59 | 59 | 57 |
| 3 months | 66 | 51 (77.3) | 51 (77.3) | 51 (77.3) | 51 (77.3) | 51 (77.3) | 51 (77.3) | 51 (77.3) | 51 (77.3) | 51 | 51 | 50 | 49 |
| 6 months | 64 | 48 (75.0) | 47 (73.4) | 48 (75.0) | 48 (75.0) | 48 (75.0) | 48 (75.0) | 48 (75.0) | 48 (75.0) | 48 | 48 | 48 | 47 |
| 12 months | 61 | 43 (70.5) | 43 (70.5) | 43 (70.5) | 43 (70.5) | 42 (68.9) | 42 (68.9) | 42 (68.9) | 42 (68.9) | 42 | 42 | 42 | 42 |
