# Supplementary material for: Emergency Laparotomy Follow-Up Study (ELFUS): prospective feasibility investigation into postoperative complications and quality of life using patient-reported outcome measures up to a year after emergency laparotomy
Source: Perioper Med (Lond). 2021 Jul 26;10:22. doi: 10.1186/s13741-021-00193-5 (PMC8311937; doi:10.1186/s13741-021-00193-5)
Supplement: Supplementary file 4 — Additional file 4:. Number of respondents to EQ5D domains at candidate follow up timepoints (portrait table only). [file 13741_2021_193_MOESM4_ESM.docx]

***Additional file 4: Number (proportion) of respondents to EQ5D domains at candidate follow up timepoints***

| Time point | Number of possible respondents (survivors) | Respondents (%) to EQ5D domains | | | | |
| --- | --- | --- | --- | --- | --- | --- |
|  |  | Mobility | Selfcare | Usual Activities | Pain | Anxiety/ Depression |
| Baseline | 70 | 70 | 70 | 70 | 70 | 70 |
| 1 month | 68 | 58 (85.3) | 58 (85.3) | 58 (85.3) | 59 (86.8) | 59 (86.8) |
| 3 months | 66 | 51 (77.3) | 51 (77.3) | 51 (77.3) | 50 (75.8) | 51 (77.3) |
| 6 months | 64 | 48 (75) | 48 (75) | 48 (75) | 48 (75) | 48 (75) |
| 12 months | 61 | 43 (70.5) | 43 (70.5) | 43 (70.5) | 43 (70.5) | 42 (68.9) |
