# Supplementary material for: Emergency Laparotomy Follow-Up Study (ELFUS): prospective feasibility investigation into postoperative complications and quality of life using patient-reported outcome measures up to a year after emergency laparotomy
Source: Perioper Med (Lond). 2021 Jul 26;10:22. doi: 10.1186/s13741-021-00193-5 (PMC8311937; doi:10.1186/s13741-021-00193-5)
Supplement: Supplementary file 5 — Additional file 5:. Additional file 5: Proportion of respondents reporting levels 1-5 in EQ5D at candidate follow up points (portrait table only). [file 13741_2021_193_MOESM5_ESM.docx]

***Additional file 5: Respondents reporting levels 1-5 in EQ5D at candidate follow up points (%)***

|  | | **Baseline** | **Day 30** | **3 months** | **6 months** | **12 months** |
| --- | --- | --- | --- | --- | --- | --- |
| **Mobility** | **Level 1** | **65.7** | **39.7** | **60.8** | **62.5** | **69.8** |
|  | **Level 2** | **5.7** | **27.6** | **17.6** | **18.8** | **7.0** |
|  | **Level 3** | **18.6** | **22.4** | **7.8** | **10.4** | **7.0** |
|  | **Level 4** | **7.1** | **5.2** | **9.8** | **6.3** | **14.0** |
|  | **Level 5** | **2.9** | **5.2** | **3.9** | **2.1** | **2.3** |
| **Self care** | **Level 1** | **70.0** | **62.1** | **72.5** | **81.3** | **81.4** |
|  | **Level 2** | **12.9** | **20.7** | **7.8** | **6.3** | **0** |
|  | **Level 3** | **11.4** | **8.6** | **11.8** | **12.5** | **11.6** |
|  | **Level 4** | **2.9** | **3.4** | **5.9** | **0** | **4.7** |
|  | **Level 5** | **2.9** | **5.2** | **2.0** | **0** | **2.3** |
| **Usual activity** | **Level 1** | **58.6** | **12.1** | **37.3** | **58.3** | **58.1** |
|  | **Level 2** | **14.3** | **29.3** | **27.5** | **18.8** | **20.9** |
|  | **Level 3** | **14.3** | **27.6** | **19.6** | **12.5** | **0** |
|  | **Level 4** | **5.7** | **13.8** | **3.9** | **6.3** | **16.3** |
|  | **Level 5** | **7.1** | **17.2** | **11.8** | **4.2** | **4.7** |
| **Pain/ discomfort** | **Level 1** | **41.4** | **35.6** | **51.0** | **60.4** | **58.1** |
|  | **Level 2** | **15.7** | **33.9** | **33.3** | **16.7** | **18.6** |
|  | **Level 3** | **22.9** | **23.7** | **7.8** | **16.7** | **18.6** |
|  | **Level 4** | **12.9** | **6.8** | **3.9** | **4.2** | **0** |
|  | **Level 5** | **7.1** | **0** | **2.0** | **2.1** | **4.7** |
| **Anxiety/ depression** | **Level 1** | **52.9** | **45.8** | **51.0** | **72.9** | **58.1** |
|  | **Level 2** | **21.4** | **22.0** | **27.5** | **10.4** | **23.3** |
|  | **Level 3** | **12.9** | **23.7** | **21.6** | **10.4** | **11.6** |
|  | **Level 4** | **4.3** | **8.5** | **0** | **4.2** | **2.3** |
|  | **Level 5** | **8.6** | **0** | **0** | **2.1** | **2.3** |
