# Supplementary material for: Emergency Laparotomy Follow-Up Study (ELFUS): prospective feasibility investigation into postoperative complications and quality of life using patient-reported outcome measures up to a year after emergency laparotomy
Source: Perioper Med (Lond). 2021 Jul 26;10:22. doi: 10.1186/s13741-021-00193-5 (PMC8311937; doi:10.1186/s13741-021-00193-5)
Supplement: Supplementary file 6 — Additional file 6:. Additional file 6: Respondents reporting EQ5D problems or no problems at candidate follow up points (portrait table only). [file 13741_2021_193_MOESM6_ESM.docx]

***Additional file 6: Respondents reporting EQ5D problems at candidate follow up points***

|  |  | **Baseline** | **Day 30** | **3 months** | **6 months** | **12 months** |
| --- | --- | --- | --- | --- | --- | --- |
| ***Mobility*** | ***No problems*** | ***46*** | ***23*** | ***31*** | ***30*** | ***30*** |
|  | ***Problems*** | ***24*** | ***35*** | ***20*** | ***28*** | ***13*** |
| ***Self care*** | ***No problems*** | ***49*** | ***36*** | ***37*** | ***39*** | ***35*** |
|  | ***Problems*** | ***21*** | ***22*** | ***14*** | ***9*** | ***8*** |
| ***Usual Activity*** | ***No problems*** | ***41*** | ***7*** | ***19*** | ***28*** | ***25*** |
|  | ***Problems*** | ***29*** | ***51*** | ***32*** | ***20*** | ***18*** |
| ***Pain/ discomfort*** | ***No problems*** | ***29*** | ***21*** | ***26*** | ***29*** | ***25*** |
|  | ***Problems*** | ***41*** | ***38*** | ***24*** | ***19*** | ***18*** |
| ***Anxiety/ depression*** | ***No problems*** | ***37*** | ***27*** | ***26*** | ***35*** | ***25*** |
|  | ***Problems*** | ***33*** | ***32*** | ***25*** | ***13*** | ***17*** |
